# Measuring breastfeeding prevalence using demographic and health surveys

**DOI:** 10.1186/s12889-024-19821-y

**Published:** 2024-08-31

**Authors:** Bastien Chabé-Ferret

**Affiliations:** 1 Middlesex University, London, UK; 2 IZA Institute of Labor Economics, Bonn, Germany

**Keywords:** Breastfeeding, Measurement, DHS, Early initiation, Exclusive breastfeeding, Breastfeeding duration

## Abstract

**Background:**

This study aims to investigate the measurement of breastfeeding prevalence indicators using Demographic and Health Surveys (DHS) data, focusing on early initiation, exclusive breastfeeding, and continued breastfeeding indicators as reported by the World Health Organization (WHO) and the United Nations Children’s Fund (UNICEF) and on the discrepancies arising from small changes in their definition.

**Methods:**

Two hundred sixty DHS samples from 78 countries were analyzed to re-calculate usual indicators reported by WHO and UNICEF: early initiation of breastfeeding (EIB), exclusive breastfeeding under 6 months (EBF), and continued breastfeeding between 1 and 2 years (CBF12 and CBF24). Additionally, alternative estimates of the same indicators, slightly changing their definition, were calculated to test their robustness.

**Results:**

The WHO and UNICEF indicators for early initiation (EIB) primarily capture cases where breastfeeding is initiated “immediately” after birth, omitting those initiated within 0 or 1 hour. This discrepancy leads to substantial underestimation of levels in some regions, particularly South Asia, and in trends. Furthermore, sizable discrepancies between exclusive breastfeeding (EBF) indicators arise from the inclusion or exclusion of plain water in the definition, with significant variations across regions, especially in West and Middle Africa. However, continued breastfeeding indicators showed consistency across definitions, proving them robust for international comparisons and time trend estimations.

**Conclusion:**

This study highlights the importance of understanding how breastfeeding indicators are defined and calculated using DHS data. Researchers should be cautious when using WHO and UNICEF indicators for early initiation and exclusive breastfeeding, as they may underestimate prevalence due to their narrow definition. Continued breastfeeding indicators, on the other hand, are less affected by small changes in definitions and provide reliable measures for cross-country comparisons and trend analyses. These findings underscore the need for standardized robust definitions and transparent reporting of breastfeeding indicators in global health assessments.

**Supplementary Information:**

The online version contains supplementary material available at 10.1186/s12889-024-19821-y.

## Background

Breastfeeding is consistently found to be associated with positive outcomes for mothers and children [1–3]. Since the late 1980s, global programs have been launched to promote early, exclusive and continued breastfeeding [4–7]. Accurate assessment of breastfeeding prevalence and trends is crucial to inform public health policies and interventions aimed at improving maternal and child well-being. Substantial data collection efforts allowed to monitor the evolution of various health indicators consistently in a large number of low and middle income countries. In particular, the Demographic and Health Surveys (DHS), funded by USAID, and the Multi-Indicator Cluster Surveys (MICS), funded by UNICEF, have used very similar questionnaires to measure (self-reported) breastfeeding. WHO and UNICEF routinely compile these data into country-year aggregates that are widely used by researchers and practitioners.

Among these indicators, three have been under particular scrutiny due to their alignment with the WHO recommendations in terms of breastfeeding. Indeed, the WHO recommends initiation of breastfeeding within one hour of birth, 6 months of exclusive breastfeeding, and continued breastfeeding until 24 months. To monitor progress on these recommendations, corresponding indicators have been developed based on the DHS/MICS questionnaire:

- Early Initiation of Breastfeeding (EIB): The share of children born in the last 24 months who were put to the breast within one hour of birth;
- Exclusive Breastfeeding under 6 months (EBF): The share of children born in the last 6 months who are currently breastfed and have not received anything else than breast milk in the last 24 hours;
- Continued Breastfeeding at 12 months (CBF): The share of children aged 12 to 15 months who have received breast milk in the previous day.

These indicators, in particular for exclusive and continued breastfeeding, have been criticised for using a 24-hour recall method and pooling together children whose age differed by several months. Exclusive breastfeeding under 6 months as measured by this method has been found to provide inflated figures compared other methods asking mothers to recall whether they were still exclusively breastfeeding when the child was 6-month old [8–12]. Nonetheless, their alignment with WHO recommendations and their consistent availability over a broad sample of countries and years ensured their success among researchers and practitioners.

An issue that has been overlooked however is the robustness of these indicators to small changes in their definition. Indeed, some discrepancies in the coding of answers may arise across countries and waves due to differences in translation or training of enumerators. It is therefore interesting to test whether modifying slightly the definition of indicators, to accommodate differences in interpretation of the questions and answers, leads to important inconsistencies in terms of cross-country and time series comparisons.

To address this issue, data from 267 DHS surveys were re-analyzed to calculate standard breastfeeding indicators, as well as alternative ones using small modifications of their definition. These recalculations were then matched and compared to indicators issued by the WHO and UNICEF. The distribution of discrepancies over time and space between indicators was then analysed, as well as their degree of correlation.

The rest of the article starts by presenting the data, then analyzes each dimension one by one, followed by a study of the correlation between indicators, a short discussion of the implications of the results together with their limitations, and finally some concluding remarks.

## The data

In February 2020, all available standard DHS data were retrieved from the official website [13]. The objective was to re-calculate key indicators related to breastfeeding practices, including early initiation, exclusive breastfeeding, and continued breastfeeding. A total of 267 data samples were collected from 78 different countries, and the details are summarized in Table A.1. However, not all samples contained the necessary information to calculate early initiation and exclusive breastfeeding indicators, as indicated in Table 1.

**Table 1:** Summary of samples included

	Early initiation		Exclusive breastfeeding		Continued breastfeeding	
	Overall	Matched	Overall	Matched	Overall	Matched
**Number of countries**	71	69	77	74	78	73
**Number of samples**	229	187	246	225	260	223

In addition, aggregate data were obtained from the UNICEF’s website [14] in both February 2020 and July 2023. Similar data were downloaded from the WHO’s website [15]. These sources were matched with the DHS data to facilitate comparisons. The matching process resulted in a somewhat reduced sample size, primarily because indicators from the initial wave of DHS surveys were not included in the WHO and UNICEF datasets. Nevertheless, the matched dataset still represented the vast majority of country-year observations, as illustrated in Figure A.1.

## Early initiation

### Which indicator do the WHO/UNICEF actually report?

Indicators related to early initiation of breastfeeding are based on the question: “How long after birth did you first put the child to the breast?” Responses are categorized as “immediately,” “within *x* hours,” or “within *y* days,” with *x* and *y* values filled in by the enumerator if needed. The indicator reported by UNICEF, denoted as EIB-unicef, measures the “Percentage of children born in the last 24 months who were put to the breast within one hour of birth.” This indicator is nearly identical to the WHO’s measurement, with only minor differences due to rounding.

From the original DHS data, the three following indicators were calculated for comparison purposes:

1. *Early Initiation of Breastfeeding (immediately) - EIB-immediate*: Percentage of children born in the last 24 months who were put to breast immediately after birth;
2. *Early Initiation of Breastfeeding (first hour) - EIB-first-hour*: Percentage of children born in the last 24 months who were put to breast either immediately after birth or within 0 or 1 hour after birth;
3. *Early Initiation of Breastfeeding (first day) - EIB-first-day*: Percentage of children born in the last 24 months who were put to breast either immediately after birth, within 0 to 24 hours after birth or within 1 day after birth.

**Fig. 1:**
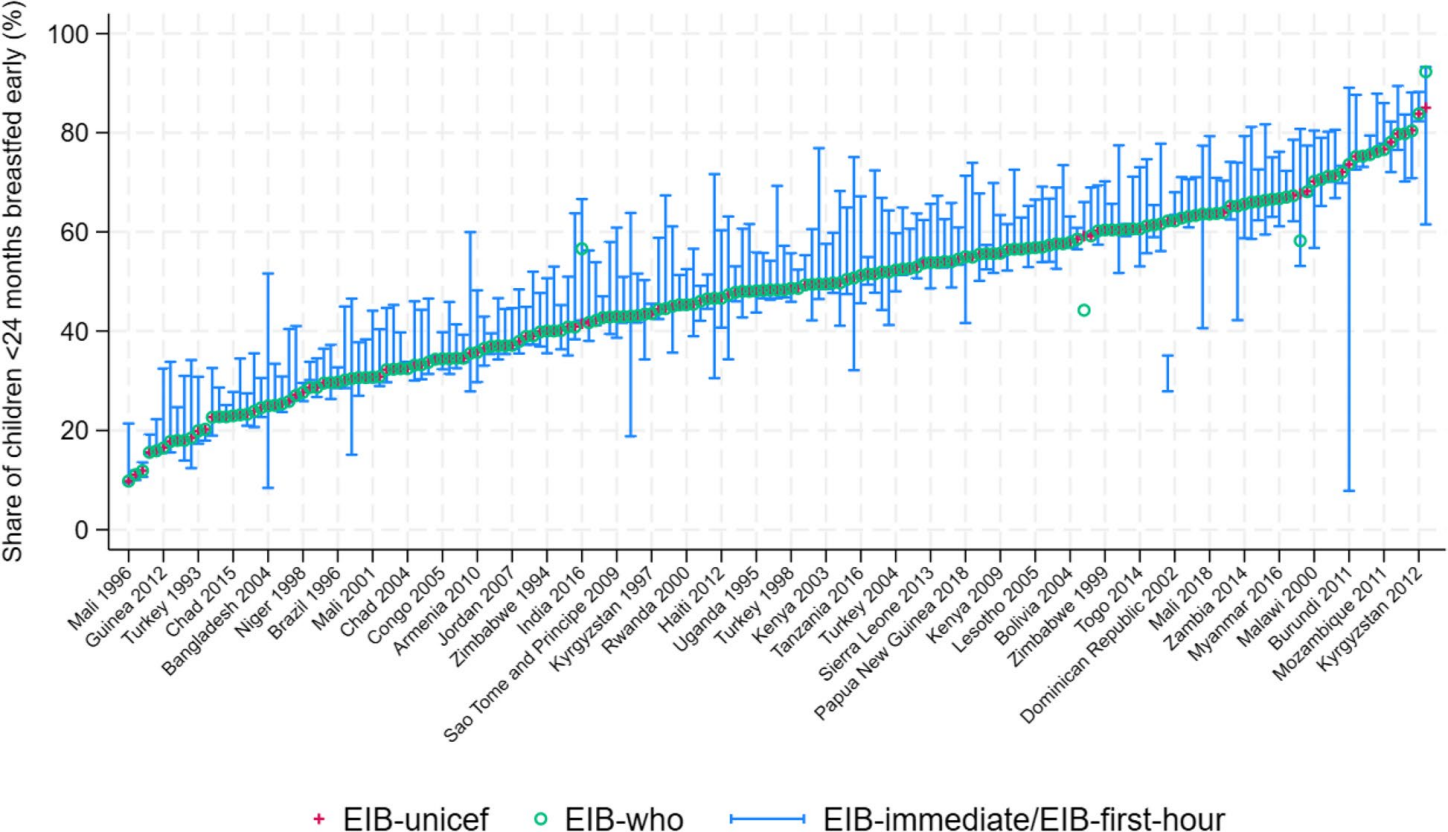
Early initiation of breastfeeding across DHS surveys and indicators * Source: DHS surveys and WHO/UNICEF*. Note: Indicators are calculated on children under 24 months

Figure 1 illustrates these indicators for each country-year pair available from the lowest to the highest prevalence of EIB-unicef (red crosses). In addition, it shows the WHO estimate (green circle) and the range going from EIB-immediate to EIB-first-hour, both re-calculated from the original DHS data (blue lines). Four main observations can be drawn from this figure:

(i) Estimates from the WHO and UNICEF are equal, except in a handful of cases;
(ii) EIB-unicef almost always lies between EIB-immediate and EIB-first-hour;
(iii) EIB-unicef lies on average much closer to EIB-immediate than EIB-first-hour;
(iv) The difference between EIB-immediate and EIB-first-hour can become non-negligible.

Table 2 presents summary statistics, showing that UNICEF’s estimate closely aligns with the indicator EIB-immediate. Indeed, on average, EIB-unicef is much closer to EIB-immediate, with only a 4 percentage point difference, than to EIB-first-hour, standing at a 9.8 percentage point distance. This indicates that the WHO and UNICEF tend to count as “early initiation” only those cases where the answer “immediately” was reported by enumerators. They consequently overlook as “early initiation” cases where responses indicated initiation within 0 or 1 hour after birth, while they technically fall within the definition of having been put to breast within one hour of birth. This introduces an error in the measurement of early initiation, as the answer “immediately”, and its translation into different languages, may be subject to interpretations, which could vary across enumerators, countries and time. The following subsection explores whether these measurement errors bias comparisons across countries and over time.

**Table 2:** Early initiation of breastfeeding - Summary Statistics

	Mean (SD)
EIB-unicef (UNICEF estimate) (%)	47.47 (16.68)
EIB-immediate (%)	43.47 (16.48)
EIB-first-hour (%)	57.27 (17.60)
EIB-immediate - EIB-first-hour difference (pp)	13.81 (9.74)
EIB-unicef - EIB-immediate difference (pp)	4.00 (6.87)
EIB-first-hour - EIB-unicef difference (pp)	9.80 (6.10)
N	187

*Source: DHS surveys and WHO/UNICEF*

“pp” stands for percentage points

### Distribution of discrepancies in indicators across time and space

**Fig. 2:**
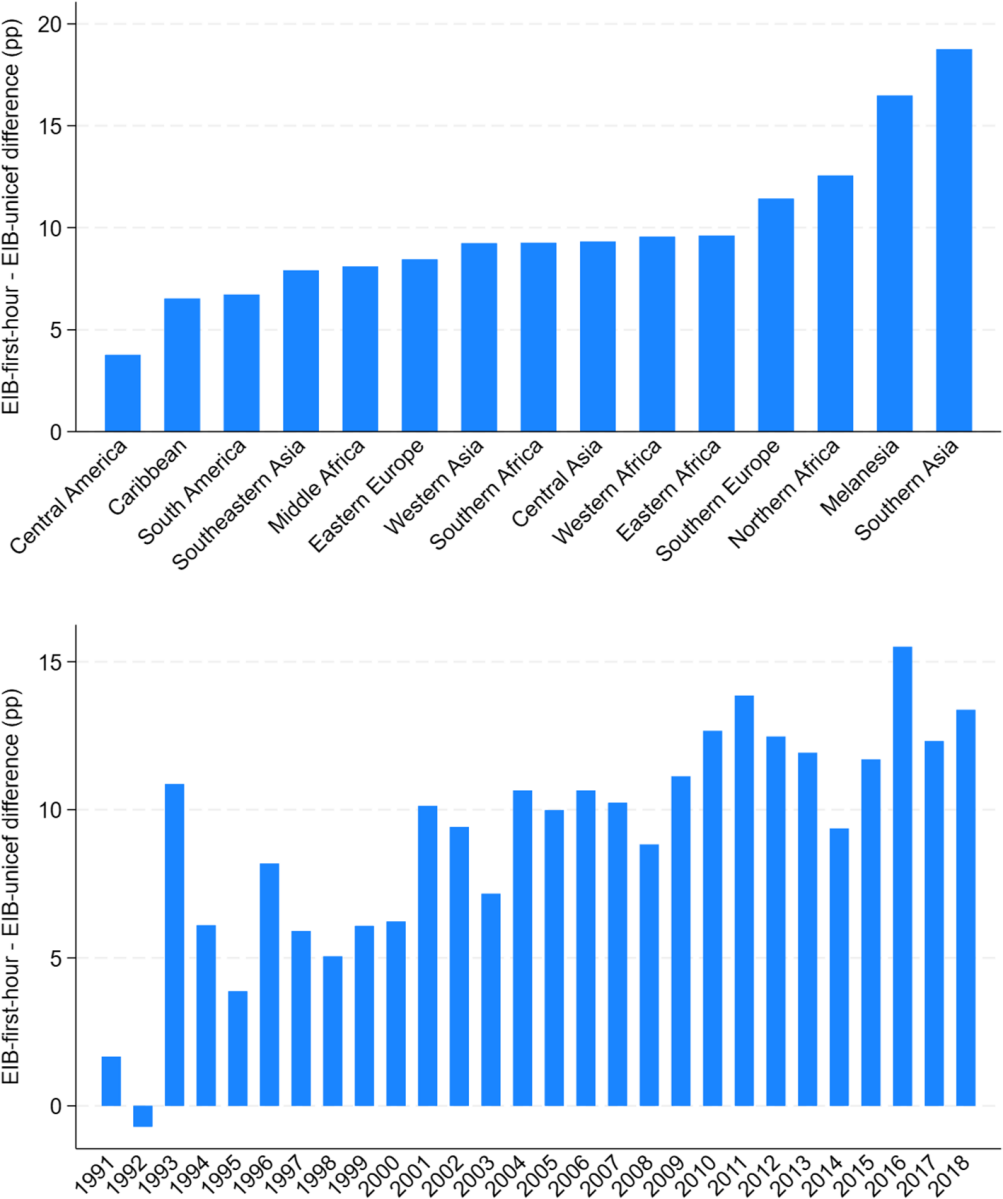
Difference between reported and re-calculated rates of Early Initiation across time and space * Source: DHS surveys and WHO/UNICEF*. Note: “pp” stands for percentage points

The top panel of Fig. 2 displays the geographical distribution of differences between the WHO/UNICEF estimates (EIB-unicef) and the recalculated indicator (EIB-first-hour). This difference is quite tightly distributed around its mean of 9.8 percentage points, but there are some notable outliers at both ends of the distribution. Early initiation seems to be the most under-reported by WHO/UNICEF for the densely populated subregion of Southern Asia, with an average discrepancy approaching 20 pp. Conversely, the reported rates tend to lie close to re-calculated ones in Central and South America as well as the Caribbean and Southeastern Asia. Any study including those subregions in an international comparison perspective may be at risk of picking up spurious correlations.

The bottom panel of Fig. 2 illustrates the increasing extent of misreporting over time, of approximately 4 percentage points per decade. Consequently, using WHO/UNICEF data to estimate time trends will tend to produce a downward bias. The magnitude of this downward bias is quantified in Table 3. The table shows first the estimated slope of a linear time trend and then the average growth rate, obtained by fitting a linear time trend to the log-transformed indicators of early initiation. To obtain those averages over the whole sample, each country has been weighed by its average population over the period 1990-2019^1^. The share of children breastfed in their first hour of life has increased by close to 1.6 percentage points per year over the last three decades in our sample, while the WHO/UNICEF estimates report an increase of only 0.95 percentage point annually, which represents a downward bias of 40% with respect to the recalculated estimate. Similarly, the average growth rate found using EIB-first-hour is 3.6% per year, while it is only 3.1% when using EIB-unicef, representing a downward bias of 14%.

**Table 3:** Difference in estimated time trends with reported and re-calculated rates of Early Initiation

	(1)	(2)	(3)	(4)
	EIB-first-hour	EIB-unicef	log(EIB-first-hour)	log(EIB-unicef)
year	1.576^***^	0.953^***^	0.036^***^	0.031^***^
	(0.348)	(0.335)	(0.010)	(0.011)
Observations	187	187	187	187
$R^{2}$	0.368	0.181	0.366	0.213

^*^p < 0.10, ^**^p < 0.05, ^***^p < 0.01. *Source: DHS surveys and WHO/UNICEF*

## Exclusive breastfeeding

### Which indicator do the WHO/UNICEF actually report?

Indicators of exclusive breastfeeding are based on questions about the child’s diet, specifically: “Are you currently breastfeeding [name of the last child]?” and “At any time yesterday or last night, was [name of last child] given any of the following: Plain water? Juice? Powdered milk? Cow’s or goat’s milk? Any other liquid? (specify) Any solid or mushy food? (specify)”. The indicator reported by UNICEF, denoted as EBF-unicef, is defined as the “Percentage of infants 0-5 months of age who are fed exclusively with breast milk”, excluding plain water but allowing vitamins, medicines, and oral rehydration solution (ORS). The WHO’s indicator is virtually identical.

From the original DHS data, the two following indicators were calculated for comparison purposes:

1. *Exclusive Breastfeeding under 6 months - EBF-strict*: Percentage of children born in the last 6 months whose mother was still breastfeeding and who did not receive any food other than breast milk;
2. *Quasi-Exclusive Breastfeeding under 6 months - EBF-quasi*: Percentage of children born in the last 6 months whose mother was still breastfeeding and who did not receive any food other than breast milk or plain water.

**Fig. 3:**
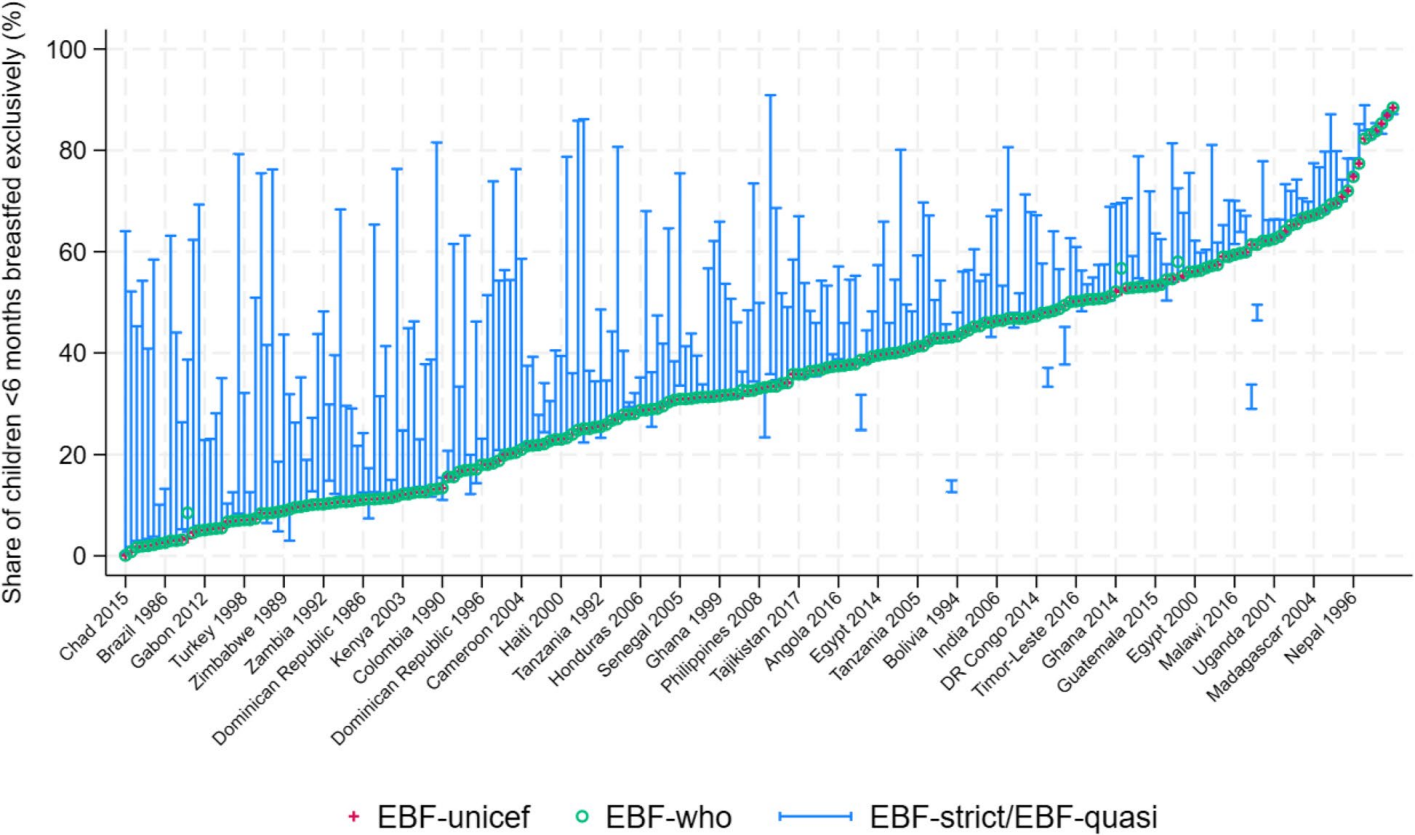
Exclusive breastfeeding across DHS surveys and indicators * Source: DHS surveys and WHO/UNICEF*. Note: Indicators are calculated on children under 6 months of age

Figure 3 displays these indicators for each country-year pair available from the lowest to the highest prevalence of EBF-unicef (red crosses). In addition, it shows the WHO estimate (green circle) and the range going from EBF-strict to EBF-quasi, both recalculated from the original DHS data (blue lines). Three main conclusions can be drawn from this figure:

(i) Estimates from the WHO and UNICEF are equal, except in a handful of cases;
(ii) Estimates from the WHO/UNICEF are extremely close to the recalculated estimate EBF-strict, except in a few cases;
(iii) Accepting plain water as part of the diet can result in substantial discrepancies.

Table 4 provides summary statistics, confirming that UNICEF’s estimate is almost identical to the re-calculated indicator EBF-strict, while the inclusion of plain water results in a substantial difference of approximately 20 percentage points. This reveals that the prevalence of children under 6 months of age being given water, while otherwise exclusively breastfed, is far from being negligible. In addition, the standard deviation of this difference is approximately 17, which suggests that there is ample variation across countries and/or over time. This introduces another source of error in the measurement of breastfeeding prevalence, as it may capture, to a large extent, differences in the risk of dehydration and/or in the awareness and reporting by parents and enumerators of ORS. The distribution of these discrepancies over time and space is documented in the next subsection.

**Table 4:** Exclusive breastfeeding - Summary Statistics

	Mean (SD)
EBF-unicef (%)	34.24 (21.14)
EBF-strict (%)	33.71 (20.92)
EBF-quasi (%)	53.27 (19.04)
EBF-strict - EBF-quasi difference (pp)	19.56 (16.63)
EBF-strict - EBF-unicef difference (pp)	-0.53 (3.72)
EBF-quasi - EBF-unicef difference (pp)	19.03 (17.74)
N	225

*Source: DHS surveys and WHO/UNICEF.*

“pp” stands for percentage points

### Exclusive and quasi-exclusive breastfeeding across time and space

**Fig. 4:**
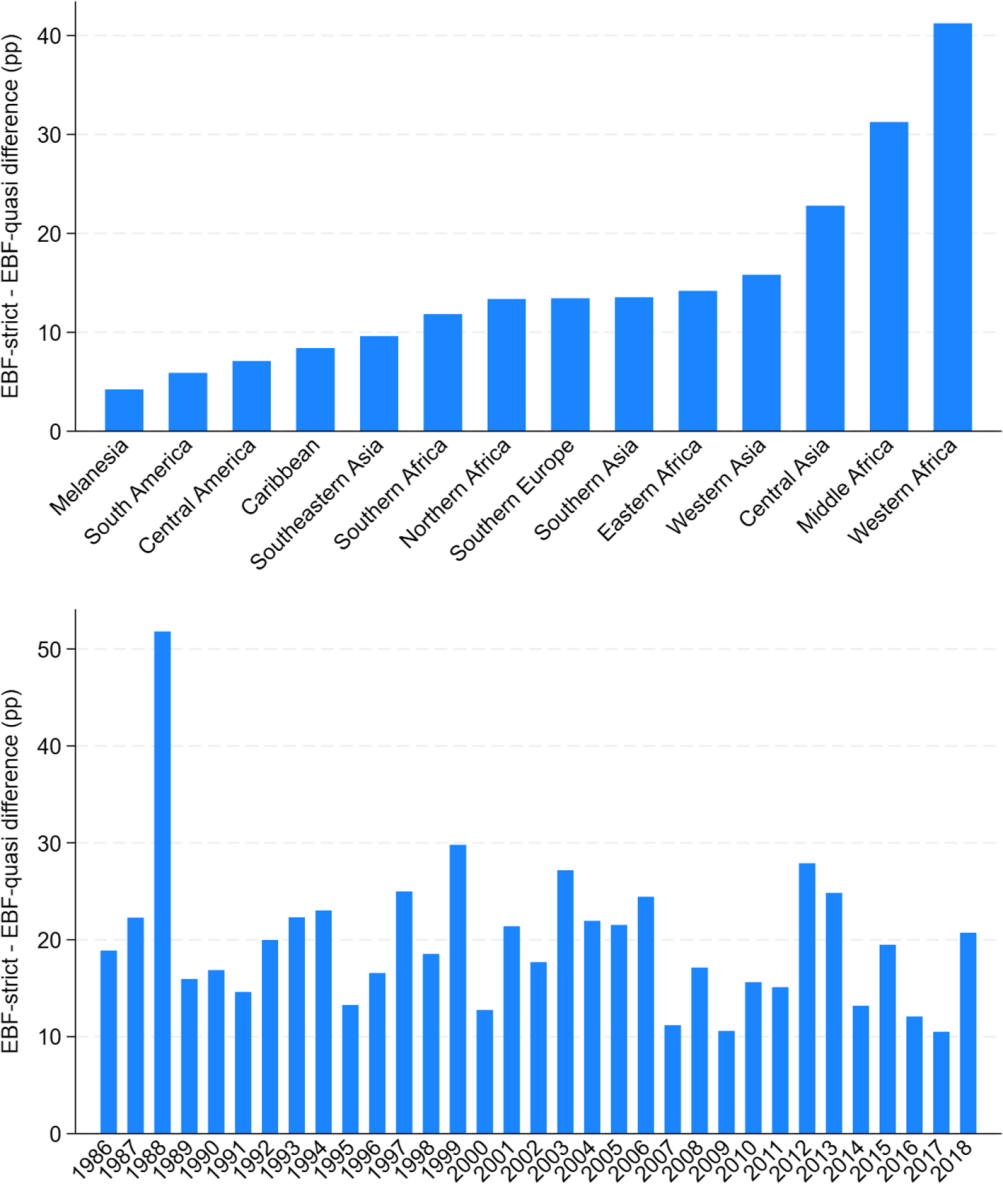
Difference between Exclusive and Quasi-Exclusive Breastfeeding across time and space * Source: DHS surveys and WHO/UNICEF*. Note: “pp” stands for percentage points

The top panel of Fig. 4 illustrates the geographical distribution of differences between exclusive and quasi-exclusive breastfeeding. Notably, those differences are small (less than 10 percentage points) in the American continent, while they are moderate (10-25 percentage points) in Asia to very large (over 30 percentage points) in some parts of Africa. One should therefore not use exclusive breastfeeding alone to make comparisons of breastfeeding prevalence across countries. Indeed, EBF-strict leads to the exclusion of many regions where breastfeeding is widely common, but where water is used too (even though not formula or other foods).

The bottom panel of Fig. 4 suggests that the difference between exclusive and quasi-exclusive breastfeeding has been declining slowly over time. Table 5 demonstrates that both exclusive and quasi-exclusive breastfeeding increased on average by 0.75 percentage point per year. However, exclusive breastfeeding shows a higher annual growth rate, 3%, compared to only 1.7% for quasi-exclusive breastfeeding, primarily due to the lower baseline prevalence of the former. The fast pace at which exclusive breastfeeding has increased over time partly reflects a decrease in the prevalence of water intake, rather than a take-up of breastfeeding.

**Table 5:** Difference in estimated time trends between Exclusive and Quasi-Exclusive Breastfeeding

	(1)	(2)	(3)	(4)
	EBF-strict	EBF-quasi	log(EBF-strict)	log(EBF-quasi)
year	0.744^***^	0.760^***^	0.031^***^	0.017^***^
	(0.200)	(0.251)	(0.010)	(0.006)
Observations	225	225	225	225
$R^{2}$	0.179	0.183	0.157	0.174

^*^p < 0.10, ^**^p < 0.05, ^***^p < 0.01. *Source: DHS surveys and WHO/UNICEF*

## Continued breastfeeding

### Which indicator do the WHO/UNICEF actually report?

Indicators for continued breastfeeding are based on questions about breastfeeding frequency during specific time periods (specifically, “last night between sundown and sun rise” and “yesterday during daylight”). UNICEF’s indicators, denoted as CBF12-unicef and CBF24-unicef, measure the “Percentage of children 12-15 months of age who are fed breast milk” and the “Percentage of children 12-23 months of age who were fed breast milk during the previous day,” respectively. The WHO’s indicator is virtually identical.

From the original DHS data and in particular the question: “Are you currently breastfeeding [name of the last child]?”, two indicators were calculated for comparison purposes:

1. *Continued Breastfeeding at 12 months - CBF12-recalc*: Percentage of children 12-15 months whose mother is still breastfeeding;
2. *Continued Breastfeeding under 24 months - CBF24-recalc*: Percentage of children 12-23 months whose mother is still breastfeeding.

**Fig. 5:**
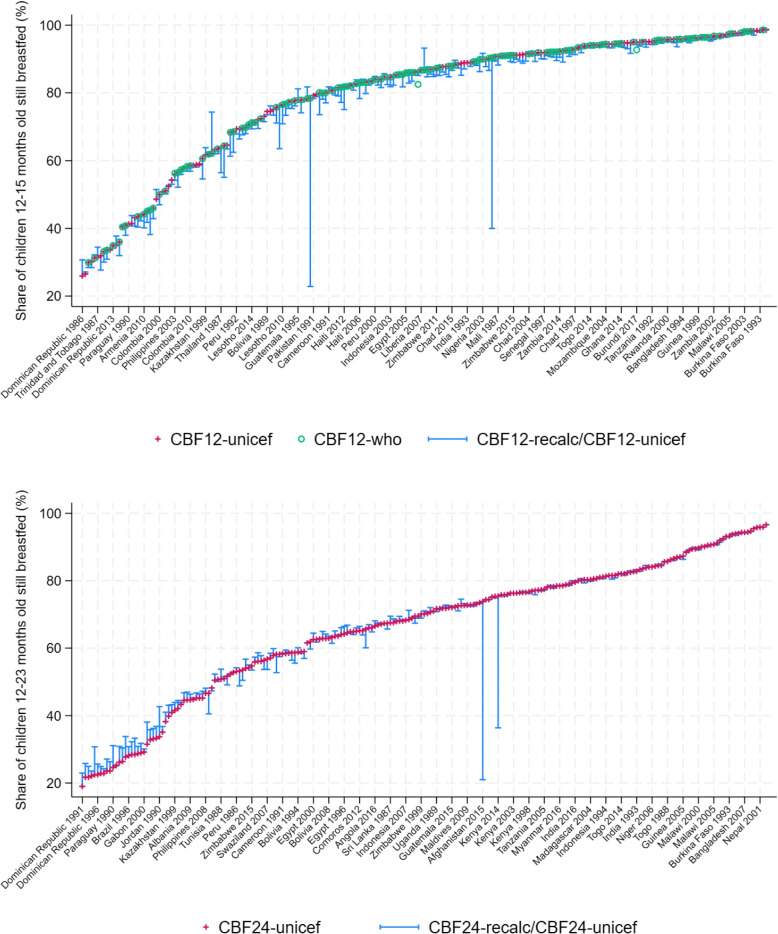
Continued breastfeeding across DHS surveys and indicators * Source: DHS surveys and WHO/UNICEF*

Figure 5 displays these indicators for each country-year pair available from the lowest to the highest prevalence of continued breastfeeding breastfeeding (red crosses). In addition, it shows the WHO estimate (green circle) and the difference between UNICEF estimates and re-calculated indicators (blue lines). Tthe three following observations can be drawn:

(i) Estimates coming from UNICEF and WHO are equal, except in a handful of cases;
(ii) Estimates from WHO/UNICEF are reasonably close to re-calculations, except in a couple of cases;
(iii) The difference between the WHO/UNICEF estimates and re-calculations tends to be larger at the lower end of the distribution.

Table 6 summarizes the statistics, indicating that UNICEF and WHO estimates closely align with the recalculated indicators CBF12-recalc and CBF24-recalc. Even though the prevalence of continued breastfeeding was calculated in a slightly different way, the differences remain negligible and are unlikely to generate spurious results. The slightly larger discrepancies at the lower end of the distribution, might reflect the greater likelihood that a mother declares that she is still breastfeeding but has actually not breastfed in the past day in a context of low prevalence of continued breastfeeding.

**Table 6:** Continued breastfeeding - Summary Statistics

	Mean (SD)
CBF12-recalc (%)	77.79 (19.76)
CBF12-unicef (%)	79.50 (19.01)
CBF12 difference(pp)	-1.71 (5.40)
CBF24-recalc (%)	66.20 (19.26)
CBF24-unicef (%)	66.09 (19.87)
CBF24 difference (pp)	0.11 (4.82)
N	223

*Source: DHS surveys and WHO/UNICEF.*

“pp” stands for percentage points

## Correlation between indicators

This section analyses the correlation between the different indicators. Breastfeeding is a multidimensional behavior, resulting from separate decisions at different points in time, taken under a set of information and constraints that also evolves over time. Nonetheless, one would expect that all these decisions are somewhat influenced by a general inclination towards breastfeeding, which would translate into some positive correlation between each dimension. Mothers initiating early should also tend to try and breastfeed exclusively, and breastfeed for the longest amount of time.

Table 7 reports the coefficient of correlation between all the re-calculated indicators over 217 DHS samples. A few observations are worth highlighting:

(i) All estimates of Early Initiation correlate with each other to a relatively high degree ($>0.7$);
(ii) EIB-immediate, which is the Early Initiation indicator closest to that reported by the WHO/UNICEF, is much less correlated to EBF-strict and not significantly correlated to EBF-quasi, while EIB-first-hour and EIB-first-day (Early Initiation within the first hour and within the first day, respectively) are strongly correlated to both EBF-strict and EBF-quasi;
(iii) None of the estimates of Early Initiation is significantly correlated to those of Continued Breastfeeding. EIB-immediate is even negatively correlated, even though not significantly so;
(iv) EBF-quasi is substantially more correlated to indicators of Continued Breastfeeding than EBF-strict is.

The weaker correlations of EIB-immediate and EBF-strict with other indicators of breastfeeding prevalence point to the possibility that they measure early initiation and exclusiveness with substantially more noise than EIB-first-hour and EBF-quasi do.

**Table 7:** Breastfeeding indicators - Correlation Matrix

	EIB-imm	EIB-1st-hour	EIB-1st-day	EBF-strict	EBF-quasi	CBF12	CBF24
EIB-immediate	1						
EIB-first-hour	0.873^***^	1					
EIB-first-day	0.702^***^	0.884^***^	1				
EBF-strict	0.286^***^	0.479^***^	0.490^***^	1			
EBF-quasi	0.0717	0.268^***^	0.339^***^	0.684^***^	1		
CBF12-recalc	-0.0708	0.0493	0.0846	0.314^***^	0.636^***^	1	
CBF24-recalc	-0.101	0.0459	0.0465	0.350^***^	0.636^***^	0.935^***^	1
*N*	217						

^*^p < 0.10, ^**^p < 0.05, ^***^p < 0.01

## Discussion

This analysis highlights the importance of understanding how indicators of breastfeeding prevalence are calculated using the DHS. Users should be warned that the indicator of early initiation reported by the WHO/UNICEF is most likely an underestimate, in particular for South Asia where the bias is large. The extent of this underestimation has risen over time, leading time trends to be downward biased.

In regard to indicators of exclusive breastfeeding, in a considerable number of cases, non-exclusivity is merely due to the ingestion of plain water. Accepting the ingestion of plain water to calculate an indicator of quasi-exclusive breastfeeding generates substantial discrepancies, especially for Western and Middle Africa. This does not mean that strictly exclusive breastfeeding should not be promoted, especially in places with poor access to safely drinkable water. This finding, however, highlights that low rates of exclusive breastfeeding should not necessarily be interpreted as indicating a low prevalence of breastfeeding. Indeed, low exclusive breastfeeding indicators are often interpreted as due to high reliance on formula, or early introduction of mushy foods. The fact that for a substantial part these low indicators are due to the use of plain water is relevant information.

Another question that arises is the familiarity of enumerators and respondents with Oral Rehydration Solution (ORS) [16]. Indeed, the definition of exclusive breastfeeding authorizes the ingestion of ORS, which is essentially plain water mixed with salts and sugar. However ORS does not appear in the possible modalities to answer the question: “Did [Name of last child] ingest any other liquid?” As documented in this paper, a non-negligible fraction of mothers respond “plain water” but another (smaller) fraction answers “sugar water”. One possibility is that indicators of exclusive breastfeeding are plagued by inconsistent reporting of ORS ingestion.

Indicators of continued breastfeeding are much more robust to small changes in the definition, and probably much more reliable for whom seeks to make international comparisons or estimate time trends.

A limitation to this analysis is the exclusive reliance on mothers’ self-reports, which may be subject to recall bias, social desirability or other types of bias. More objective measures are of course desirable, but hard to implement on large scale samples. This work actually tests the robustness of self-reported indicators to assess their reliability. Another limitation comes from the failure to include MICS samples, due to the numerous variations in variable names across countries and waves, which made the coding vastly more complex. Including them would allow a better representation of middle-income countries. A final limitation common to all breastfeeding measurement attempts using DHS is the total absence of high-income countries. The adoption of a similar questionnaire in existing surveys of infants for instance would be of great help to the research community.

## Conclusion

This article documents that small changes in the definition of the indicators reported by the WHO and UNICEF can generate large discrepancies, in particular for early initiation and exclusive breastfeeding, casting doubt on international comparisons and analysis of time trends. To document these discrepancies, data coming from 260 DHS samples were re-analyzed and matched with aggregates compiled by the WHO and UNICEF. Indicators of early initiation, exclusive and continued breastfeeding, were re-calculated and found to match closely with those computed by the WHO and UNICEF. Slightly different indicators were then created for each dimension to test their robustness. In particular, the definition of early initiation was relaxed to allow for children to have been put to breast within 0 or 1 hour after birth, and quasi-exclusive breastfeeding was defined as ingestion in the previous 24 hours of breast milk or plain water.

UNICEF/WHO estimates were found to substantially underestimate both early initiation and exclusive breastfeeding, while no such bias is found for continued breastfeeding. The underestimation of early initiation is most pronounced for South Asia and has increased over time. For exclusive breastfeeding, the underestimation is particularly large for Middle and Western Africa, and has slightly decreased over time.

This work contributes to the literature on the measurement of breastfeeding prevalence [17–19]. It sheds light on the sensitivity of usual indicators to slight modifications of their definitions. It suggests to be cautious when interpreting cross-section comparisons and time trends and to test the robustness of the results using alternative indicators such as “initiation within one hour after birth” and “quasi-exclusive breastfeeding”. These findings may have crucial implications for the large literature on the determinants and consequences of breastfeeding prevalence [20–26].

### Supplementary Information

Supplementary Material 1.

## Data Availability

$\cdot$ https://dhsprogram.com/data $\cdot$ https://www.who.int/data/nutrition/nlis/data-search $\cdot$ https://data.unicef.org/topic/nutrition/infant-and-young-child-feeding/ $\cdot$ https://data.worldbank.org/indicator/SP.POP.TOTL.

## Footnotes

1 The data was taken from the World Bank https://data.worldbank.org/indicator/SP.POP.TOTL).

## Data availability

The data for the DHS surveys is accessible from https://dhsprogram.com/data/available-datasets.cfm after registration. The data from UNICEF is accesible from https://data.unicef.org/topic/nutrition/infant-and-young-child-feeding/. The data from the WHO is accessible from https://www.who.int/data/nutrition/nlis/data-search. A replication package containing the code to calculate all indicators used in the paper and replicate all the figures and table is available upon request.
