# Supplementary material for: Measuring breastfeeding prevalence using demographic and health surveys
Source: BMC Public Health. 2024 Aug 31;24:2366. doi: 10.1186/s12889-024-19821-y (PMC11365256; doi:10.1186/s12889-024-19821-y)
Supplement: Supplementary file 1 — Supplementary Material 1. [file 12889_2024_19821_MOESM1_ESM.pdf]

Appendix A. Supplementary Tables and Figures

Figure A.1: DHS samples included

Country name	Years
Afghanistan	2015
Albania	2009, 2018[c]
Angola	2007[a,b,c], 2016
Armenia	2000, 2005[b], 2010, 2016
Azerbaijan	2006
Bangladesh	1994[a], 1997[a], 2000[a,b], 2004[b], 2007, 2011, 2014
Benin	1996[a], 2001, 2006, 2012, 2018[c]
Bolivia (Plurinational State of)	1989[a], 1994, 1998, 2004, 2008
Brazil	1986[a], 1992[a,b,c], 1996[b]
Burkina Faso	1993[a], 1999[a], 2003, 2010
Burundi	1987[a], 2011, 2013[a,b,c], 2017
Cameroon	1991, 1998, 2004, 2011
Central African Republic	1995
Chad	1997, 2004[b], 2015
Colombia	1986[a], 1990[a], 1995, 2000, 2005, 2010, 2016[a,b,c]
Comoros	1996, 2012
Congo	2005[b], 2012
Congo (Democratic Republic of the)	2007, 2014
Côte d'Ivoire	1994, 1999, 2012
Dominican Republic	1986[a], 1991[a], 1996, 1999[b,c], 2002, 2007, 2013
Ecuador	1987[a,b], 2008[a,c]
Egypt	1898[a,b], 1993[a], 1996, 2000, 2003[a,b,c], 2005[b], 2008, 2014
Ethiopia	1992[a,b,c], 1997[a,b,c], 2003[a,b,c], 2008[a,b,c]
Gabon	2000, 2012
Gambia	2013
Ghana	1988[a], 1994, 1999, 2003, 2008, 2014
Guatemala	1987[a,b], 1995, 2015
Guinea	1999, 2005, 2012, 2018[c]
Guyana	2009
Haiti	1995, 2000, 2006, 2012, 2017[c]
Honduras	2006, 2012
India	1993[a], 2000, 2006, 2016
Indonesia	1987[a,b], 1991[a], 1994[a], 1997[a], 2003, 2007, 2012, 2017[c]
Jordan	1990[a], 1997, 2002, 2007, 2012, 2018[c]
Kazakhstan	1995[c], 1999
Kenya	1989[a], 1993[a], 1998, 2003, 2009, 2014
Kyrgyzstan	1997[c], 2012
Lesotho	2005, 2010, 2014
Liberia	1986[a], 2007,2013
Madagascar	1992[a], 1997, 2004, 2009, 2011[a,b,c], 2013[a,b,c], 2016[a,b,c]
Malawi	1992[a,b], 2000, 2005, 2010[b], 2016
Maldives	2009, 2017[c]
Mali	1987[a], 1996, 2001, 2006, 2013[a,b,c], 2018[c]
Mexico	1987[a,c]
Moldova (Republic of)	2005[b]
Morocco	1987[a], 1992[a], 2004
Mozambique	1997, 2004, 2011
Myanmar	2016
Namibia	1992[a], 2000, 2007, 2013
Nepal	1996, 2001, 2006, 2011, 2016
Nicaragua	1998, 2001
Niger	1992, 1998, 2006, 2012
Nigeria	1990[a], 2003, 2008, 2013
Pakistan	1991[a], 2007[b], 2013
Papua New Guinea	2018
Paraguay	1990[a]
Peru	1986[a], 1992[a], 1996, 2000, 2008[a,b,c], 2010[a,b,c], 2012[a,b,c]
Philippines	1993[a], 1998, 2003, 2008, 2013[b], 2017[b,c]
Rwanda	1992[a], 2000, 2005, 2011, 2015
Sao Tome and Principe	2009
Senegal	1986[a], 1993[a], 1997[a], 2005, 2009[a,b,c], 2011[a,b,c], 2013[a,b,c], 2015[a,b,c], 2017[a,b,c]
Sierra Leone	2008, 2013
South Africa	1998
Sudan	1990[a,b,c]
Sri Lanka	1987[a]
Swaziland	2007
Tajikistan	2012, 2017[c]
Tanzania, United Republic of	1992[a], 1996, 1999[a], 2005, 2010, 2016
Thailand	1987[a]
Timor-Leste	2010, 2016[c]
Togo	1988[a], 1998, 2014
Trinidad and Tobago	1897[a,b]
Tunisia	1988[a]
Turkey	1993[c], 1998[c], 2004[b,c]
Uganda	1989[a], 1995, 2001, 2006, 2011, 2016
Yemen	1992[a,c], 2013
Zambia	1992[a], 1997, 2002, 2007, 2014
Zimbabwe	1989[a], 1994, 1999, 2006, 2011, 2015

a: missing from the matched early initiation sample; b: missing from the matched exclusive breastfeeding sample; c: missing from the matched continued breastfeeding sample.
